# Expression of Enhancer-Binding Protein CEBPA mRNA and Protein in Ovarian Cancer and Its Relationship With Pathobiological Characteristics

**DOI:** 10.3389/fsurg.2022.842823

**Published:** 2022-02-23

**Authors:** Shufang Mi, Limei Zhang, Mo Li, Zhiting Dong, Chenchen Tian, Minwen Fu

**Affiliations:** ^1^Gynaecology and Obstetrics, Affiliated Hospital of Beihua University, Jilin City, China; ^2^Gynaecology and Obstetrics, Jilin City Maternity Hospital, Jilin City, China

**Keywords:** ovarian cancer, CCAAT enhancer-binding protein α, protein, pathobiological characteristics, prognosis

## Abstract

Ovarian cancer is a common malignant tumor, its early onset is hidden, lack of specific symptoms, the location of the lesion is particularly hidden, which makes it difficult to find ovarian lesions by general detection, making it difficult to make an early clinical diagnosis. Therefore, it is still the focus and difficulty of ovarian cancer research to find the means of early diagnosis and prognosis of ovarian cancer. Cytosine-cytosine-adenosine-adenosine-thymidine (CCAAT) enhancer-binding protein α (CEBPA) has been proved to be involved in cell metabolism, proliferation, and differentiation. In this study, the expression of CEBPA mRNA and protein in normal ovary, epithelial ovarian cyst, ovarian borderline tumor, and ovarian cancer was detected, the relationship between CEBPA and pathobiological characteristics of ovarian cancer was discussed, and its influence on the prognosis of patients with ovarian cancer was analyzed. The results showed that the expression of CEBPA mRNA and protein in patients with ovarian borderline tumor and ovarian cancer is high, and the expression of CEBPA has no obvious correlation with the pathobiological characteristics of patients with ovarian cancer, and the high expression of CEBPA has an important value in the diagnosis of ovarian cancer, and it is also a poor prognostic factor of the disease.

## Introduction

Ovarian cancer is closely related to factors, such as heredity and hormone level. It is a complicated process that involves many factors, genes, and steps, and the peak period of ovarian cancer is over 45 years old (1). The clinical manifestations of ovarian cancer are mostly pain, and some patients may be accompanied by lower abdominal mass, irregular menstruation, etc. According to the global cancer report, in 2020, 4.43 million cases of cancer deaths among women worldwide, i.e., 210,000 cases of ovarian cancer (2). The main features of ovarian cancer are high morbidity and mortality, early-onset concealment, lack of specific symptoms, rapid progression, and high malignancy (3, 4). In addition, the ovary is deep in the pelvic cavity, with a special shape, particularly, hidden lesion location and diverse histological types. Therefore, it is difficult to detect ovarian lesions in general detection, making early clinical diagnosis more difficult (5). Cytoreductive surgery and adjuvant chemotherapy are common methods for the treatment of ovarian cancer, which are effective for more than 80% of patients with ovarian cancer and have achieved remarkable curative effects. However, due to the serious drug resistance of chemotherapy drugs, the recurrence rate of patients after treatment is high and the prognosis is not good (6). Therefore, we need to take measures to improve patient outcomes.

Cytosine-cytosine-adenosine-adenosine-thymidine/enhancer-binding proteins (C/EBPs) is a transcriptional regulatory factor of eukaryotic cells discovered by Graves' team in 1987. The C/EBP family has six members, C/EBPα, C/EBPβ, C/EBPγ, C/EBPδ, C/EBPε and C/EBPζ, and C/EBP α [CCAAT enhancer-binding protein α (CEBPA)], that contains 358 amino acids and is encoded by the CEBPA gene, with a total length of 3,318 bp and no intron (7). This transcription factor can inhibit cell proliferation by interacting with other proteins or regulating the chromatin remodeling complex. At the same time, CEBPA is involved in the regulation of hematopoiesis and the terminal differentiation of adipocytes, different epithelial cells, and other types of cells (8, 9). CEBPA not only balances cell proliferation and differentiation but also regulates cell metabolism, inhibits the process of the cell cycle, and participates in the mitosis of many cell lines. These functions can well-show the cell growth inhibition activity of CEBPA (10, 11).

Up to now, there has been no report describing how CEBPA is expressed in ovarian cancer tissues, and how the change of its expression affects the occurrence, prognosis of ovarian cancer. By detecting the expression of CEBPA mRNA and protein in ovarian cancer, the relationship between CEBPA level and clinicopathological features of ovarian cancer and its influence on the prognosis of patients were analyzed, so as to provide some basis for guiding the clinical treatment of ovarian cancer.

## Materials and Methods

### Research Object

From June 2015 to June 2018, 14 cases of normal ovary patients with non-ovarian diseases who received gynecological surgery, 38 cases of epithelial ovarian cyst, 10 cases of ovarian borderline tumor, and 71 cases of ovarian cancer in our hospital were selected. The mean age of the research object was (52.94 ± 5.18) years, there was no difference in age between the four groups. Inclusion criteria were as follows: confirmed by pathological examination; patients were scheduled for surgical treatment, and the complete lesion tissue/paracancerous tissue was obtained during the operation, but no radiotherapy, chemotherapy, and biological immunotherapy were performed before operation; Cognitive function was normal; clinical data were complete. Exclusion criteria were as follows: complicated with serious organic diseases; combined with other malignant tumors; complicated with immune system diseases; coagulation dysfunction; and mental disorder.

### Research Methods

#### Tissue Collection

Normal ovary, epithelial ovarian cyst, ovarian borderline tumor, and ovarian cancer tissues were cut out during operation, then frozen in liquid nitrogen for 30 min and stored in −80°C refrigerator. Part of the tissue was fixed with 10% formaldehyde, then embedded in paraffin, 4-μm thick sections were prepared for later use.

#### Cytosine-Cytosine-Adenosine-Adenosine-Thymidine Enhancer-Binding Protein α mRNA Detection

Total RNA was extracted from frozen samples with TRIZOL reagent. According to the instructions of the reverse transcription PCR (RT-PCR) kit, RT reaction was carried out with corresponding RT primers to synthesize the first strand of cDNA. The conditions were as follows: 42°C for 60 min, 99°C for 5 min, 4°C for 5 min, and −20°C for storing the first strand of cDNA. Real-time fluorescence quantitative PCR was used for amplification, pre-denaturation at 95°C for 3 min, then 94°C for 30 s, 72°C for 30 s, totally 23–31 cycles, prolonged at 72°C for 10 min, stored at 16°C, and put into 1.5% agarose gel electrophoresis to detect PCR products. The experiment followed the corresponding kit instructions, and the primers are shown in Table 1. GAPDH was used as an endogenous reference, and expression was calculated using 2^−Δ*ΔCt*^, compared the level of CEBPA mRNA in different tissues.

**Table 1:** Primer sequence.

**Primer**	**Forward (5^′^ → 3^′^)**	**Reverse (5^′^ → 3^′^)**
CEBPA	AACACGAAGCACGATCAGTCC	CTCATTTTGGCAAGTATCCGA
GAPDH	TGTTGCCATCAATGACCCCTT	CTCCACGACGTACTCAGCG

#### Detection of CEBPA Protein

The western blotting (WB) was used to detect the total protein. After the total protein was extracted with radioimmunoprecipitation assay (RIPA) buffer containing a protease inhibitor, the concentration of protein was measured by a decanoic acid protein assay kit. An equal amount of protein was separated by 10% sodium dodecyl sulfate-polyacrylamide gel electrophoresis (SDS-PAGE). Then, the protein was transferred to the polyvinylidene fluoride film at a constant voltage of 80 V. Five percent skim milk was used to seal the solution for 1 h, then primary antibody CEBPA (1:100) and GAPDH were added and incubated at 4°C by gently shaking overnight, washed with buffer solution (TBST) at room temperature for 3 times. The next day, the second biotin-labeled goat anti-rabbit immunoglobulin G (IgG) was oscillated at 37°C for 2 h. TBST was used at room temperature for 3 times. The polyvinylidene fluoride film was colored and photographed in a dark environment, taking GAPDH as internal control.

#### Positive Expression of CEBPA

The positive expression of CEBPA was detected by immunohistochemistry. The prepared paraffin slices were baked at 60°C for 2 h, dewaxed, and rehydrated. Then incubated with 0.3% H_2_O_2_ formaldehyde solution at room temperature for 30 min. The antigen was repaired in citric acid buffer under high pressure for 2 min. After waiting for the temperature to be the same as room temperature, washed with phosphate-buffered saline (PBS) buffer for three times. The primary antibody was diluted with antibody diluent and incubated at 4°C. The next morning, the box containing slices was taken out. The secondary antibody was dropped and incubated at room temperature for 1 h. 3,3′-Diaminobenzidine (DAB) was used for color rendering, observed under a microscope, and the color rendering was terminated in time. After the color rendering was completed, distilled water was washed, hematoxylin was re-dyed for 20 s, alcohol was dehydrated with different concentration gradients, xylene was transparent, and neutral gum was sealed. The stained sections were observed under a microscope.

Immunohistochemical results showed that the cells with brown or brown granules in the cytoplasm were positive cells by immunohistochemical staining. The semi-quantitative integral method was used to judge the expression, and the percentage of staining intensity and positive cells was recorded as 0–3 points, respectively. The staining intensity score was: 0 points (no staining), 1 point (light staining), 2 points (moderate staining), and 3 points (deep staining). The positive cell count score was as follows: 10 visual fields were randomly selected and the average positive percentage was calculated. 0 points (the number of positive cells <10%), 1 point (10–24%), 2 points (25–50%), 3 points (>50%). The scoring standard for protein staining results was determined by multiplying the staining intensity by the number of positive cells. The staining results were recorded as 0 points (–), 1–3 points (+), 4–6 points (++), and 7–9 points (+++). Staining results (–) were regarded as negative, and staining results (+), (++), and (+++) were regarded as positive. All tissue specimens were independently evaluated by two pathologists, discrepant results were evaluated by a third physician, and a consistent result was chosen for the final evaluation.

### Observation Index

The clinicopathological data of patients with ovarian cancer were collected, such as age, the International Federation of Gynecology and Obstetrics (FIGO) stage, histologic type, degree of histological differentiation, and lymph node metastasis, to compare CEBPA expression in patients with different types of ovarian cancer. The receiver operating curve (ROC) curve was drawn to analyze the diagnostic efficacy of CEBPA levels in ovarian cancer patients. Follow-up work will be completed in June 2021. During this period, ovarian cancer patients were followed up by telephone, letters, and hospital records to record their survival. Overall survival was defined as the time from the day of surgery to death or loss of follow-up.

### Statistical Methods

The Statistical Product and Service Solutions (SPSS) 22.0 software was adopted, the measurement data were expressed as (±s), the Student-Newman-Keuls (SNK)-q method was used for paired comparison, and variance analysis was used for multiple group comparisons. The counting data were expressed by rate (%), and the comparison between groups adopts the χ^2^ test. The ROC curve was constructed, and the area under the curve (AUC) was calculated to evaluate the diagnostic efficacy of CEBPA level for ovarian cancer. Kaplan-Meier survival curve was used to analyze the prognosis of patients. *P* < 0.05 means the difference was statistically significant.

## Results

### Comparison of CEBPA mRNA Expression in Ovarian Tissues of Four Groups of Patients

**Figure 1:**
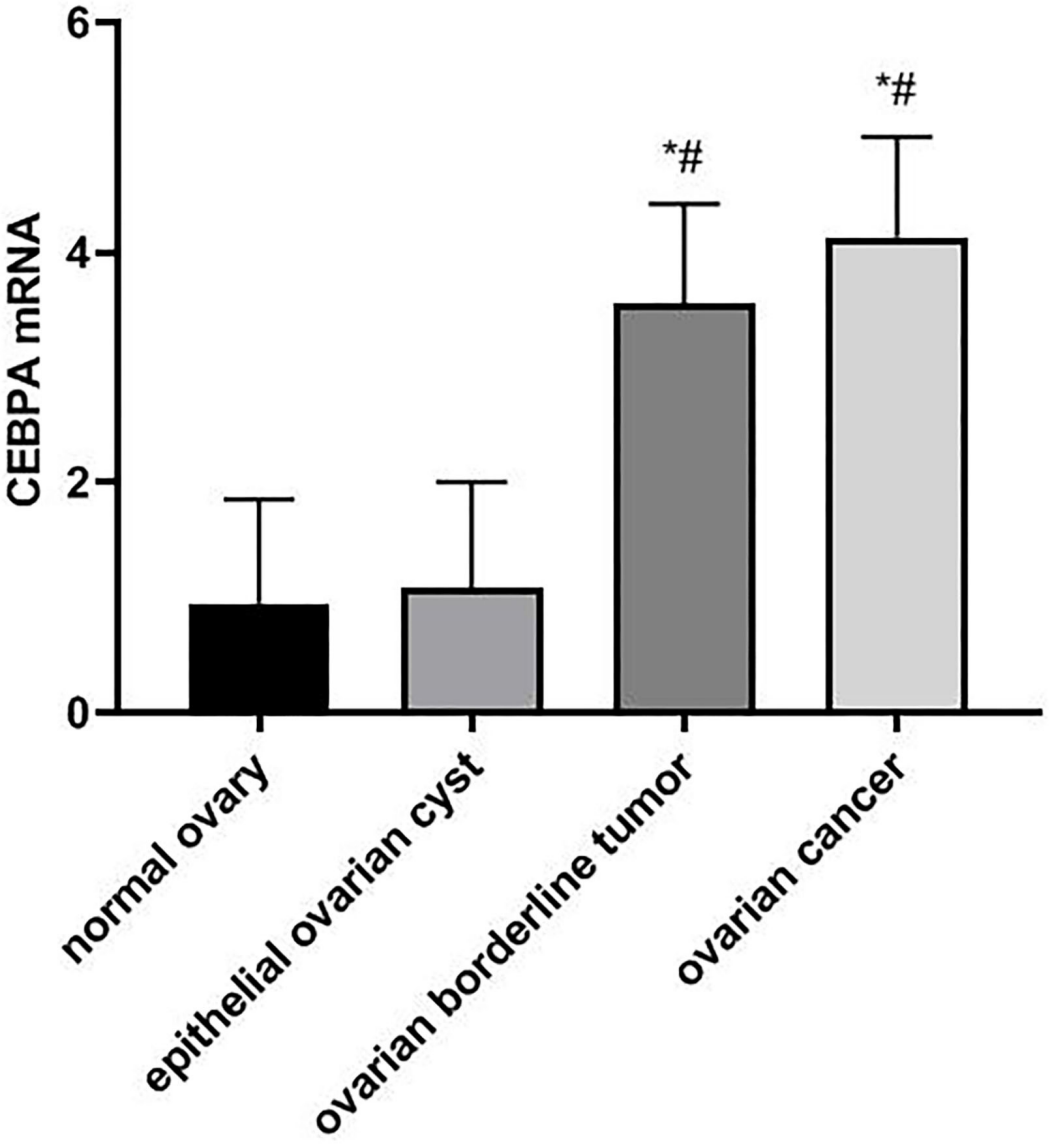
Comparison of CEBPA mRNA expression in ovarian tissues of four groups of patients. Compared with normal ovary, *p < 0.05; Compared with an epithelial ovarian cyst, #*p* < 0.05. CEBPA, CCAAT enhancer-binding protein alpha.

The results of real-time quantitative PCR showed that the expressions of CEBPA mRNA in ovarian tissues of patients with normal ovary, epithelial ovarian cyst, ovarian borderline tumor, and ovarian cancer were 0.95 ± 0.90, 1.08 ± 0.92, 3.56 ± 0.86, and 4.13 ± 0.87, respectively. Compared with normal ovary and epithelial ovarian cysts, the expression level of CEBPA mRNA in patients with ovarian borderline tumor and ovarian cancer was significantly higher (*F* = 123.223, *p* < 0.05), as shown in Figure 1.

### Comparison of CEBPA Protein Expression in Ovarian Tissues of Four Groups of Patients

**Figure 2:**
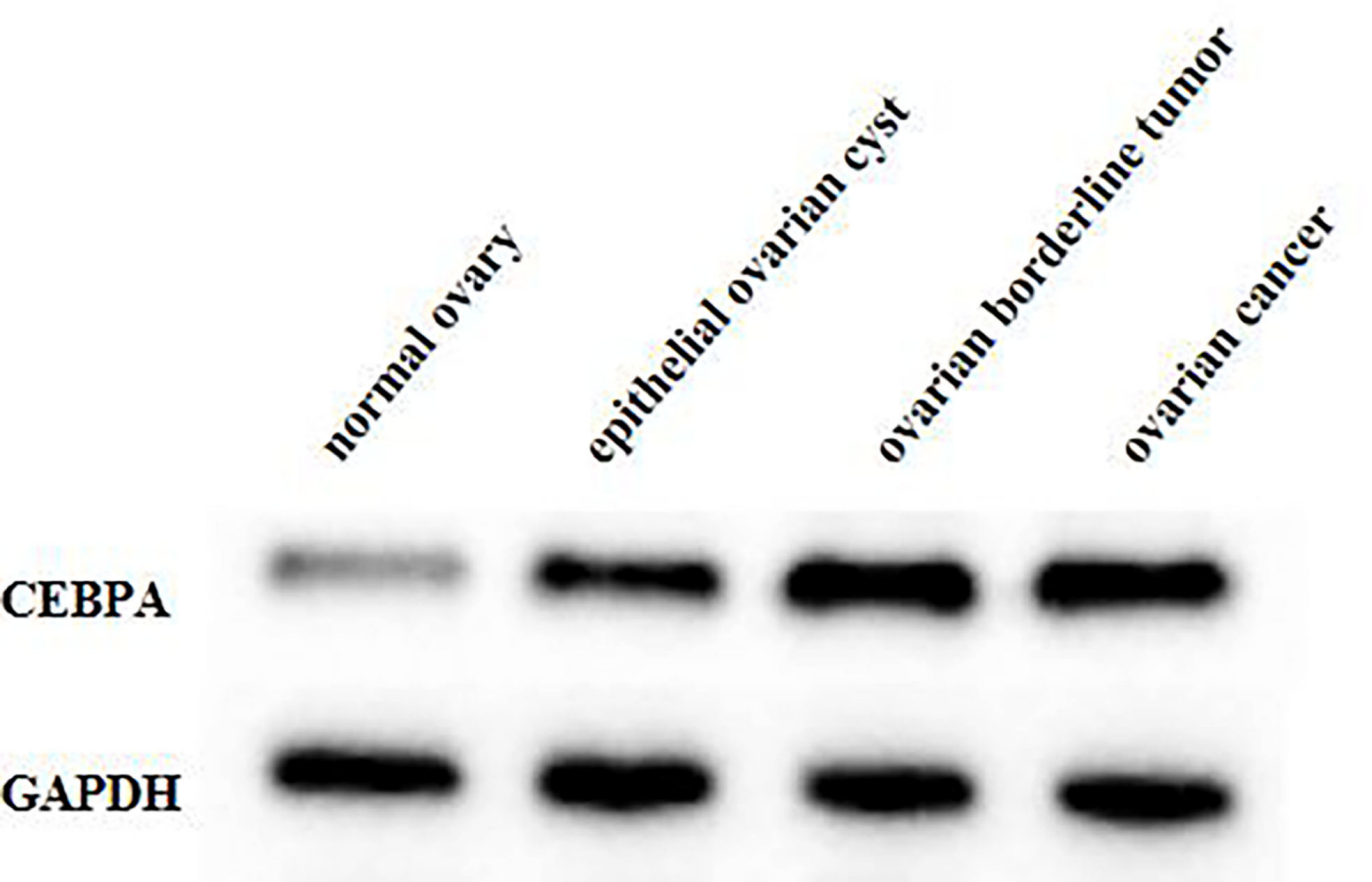
Comparison of CEBPA protein expression in ovarian tissues of four groups of patients. CEBPA, CCAAT enhancer-binding protein alpha.

The result of the WB experiment showed that the expression level of CEBPA protein in patients with ovarian borderline tumor and ovarian cancer was significantly higher than that in patients with normal ovary and an epithelial ovarian cyst (*p* < 0.05), as shown in Figure 2.

### The Positive Expression Rate of CEBPA in Different Ovarian Tissues

In normal ovarian and epithelial ovarian cysts, the positive expression rate of CEBPA was low. However, in ovarian borderline tumor and ovarian cancer tissues, the number of positive cells of CEBPA was significantly increased, and the staining was significantly deepened, and the positive expression rate was significantly higher than that of normal ovary and epithelial ovarian cyst tissues (χ^2^ = 34.932, *p* < 0.05), as shown in Table 2.

**Table 2:** Positive expression rate of CEBPA in different ovarian tissues.

**Group**	**Case number**	**Expression of CEBPA protein**				**Positive expression rate**
		**–**	**+**	**++**	**+++**	
Normal ovary	14	13	1	0	0	7.14%
Epithelial ovarian cyst	38	28	8	2	0	26.32%
Ovarian borderline tumor	10	5	1	2	2	50.00%
Ovarian cancer	71	22	24	18	7	69.01%

*CEBPA, CCAAT enhancer-binding protein alpha*.

### The Relationship Between the Expression of CEBPA and the Pathobiological Characteristics of Ovarian Cancer Patients

There was no significant correlation between the expression of CEBPA and the age, FIGO stage, histologic type, degree of histological differentiation, and lymph node metastasis of ovarian cancer patients (*p* > 0.05), as shown in Table 3.

**Table 3:** Relationship between the expression of CEBPA and the pathobiological characteristics of ovarian cancer patients [*n* (%)].

**Project**	**Total number of cases (*n* = 71)**	**CEBPA negative expression group (*n* = 22)**	**CEBPA positive expression group (*n* = 49)**	***χ^2^* value**	***P*-value**
Age				0.280	0.596
<50 years old	29	10 (45.45%)	19 (38.78%)		
≥50 years old	42	12 (54.55%)	30 (61.22%)		
FIGO stage				0.126	0.723
I,II	28	8 (36.36%)	20 (40.82%)		
III,IV	43	14 (63.64%)	29 (59.18%)		
Histologic type				1.656	0.647
Serous cystadenocarcinoma	32	10 (45.45%)	22 (44.90%)		
Mucosal cystadenocarcinoma	11	5 (22.73%)	6 (12.24%)		
Endometrial carcinoma of ovary	25	6 (27.27%)	19 (38.78%)		
Clear cell carcinoma	3	1 (4.55%)	2 (4.08%)		
Degree of histological differentiation				4.553	0.103
Highly differentiated	15	8 (36.36%)	7 (14.29%)		
Middle differentiation	42	11 (50.00%)	31 (63.27%)		
Poorly differentiated	14	3 (13.64%)	11 (22.45%)		
Lymph node metastasis				0.006	0.937
Without	35	11 (50.00%)	24 (48.98%)		
With	36	11 (50.00%)	25 (51.02%)		

*CEBPA, CCAAT enhancer-binding protein alpha*.

### Diagnostic Efficacy of CEBPA Level in Ovarian Cancer

**Figure 3:**
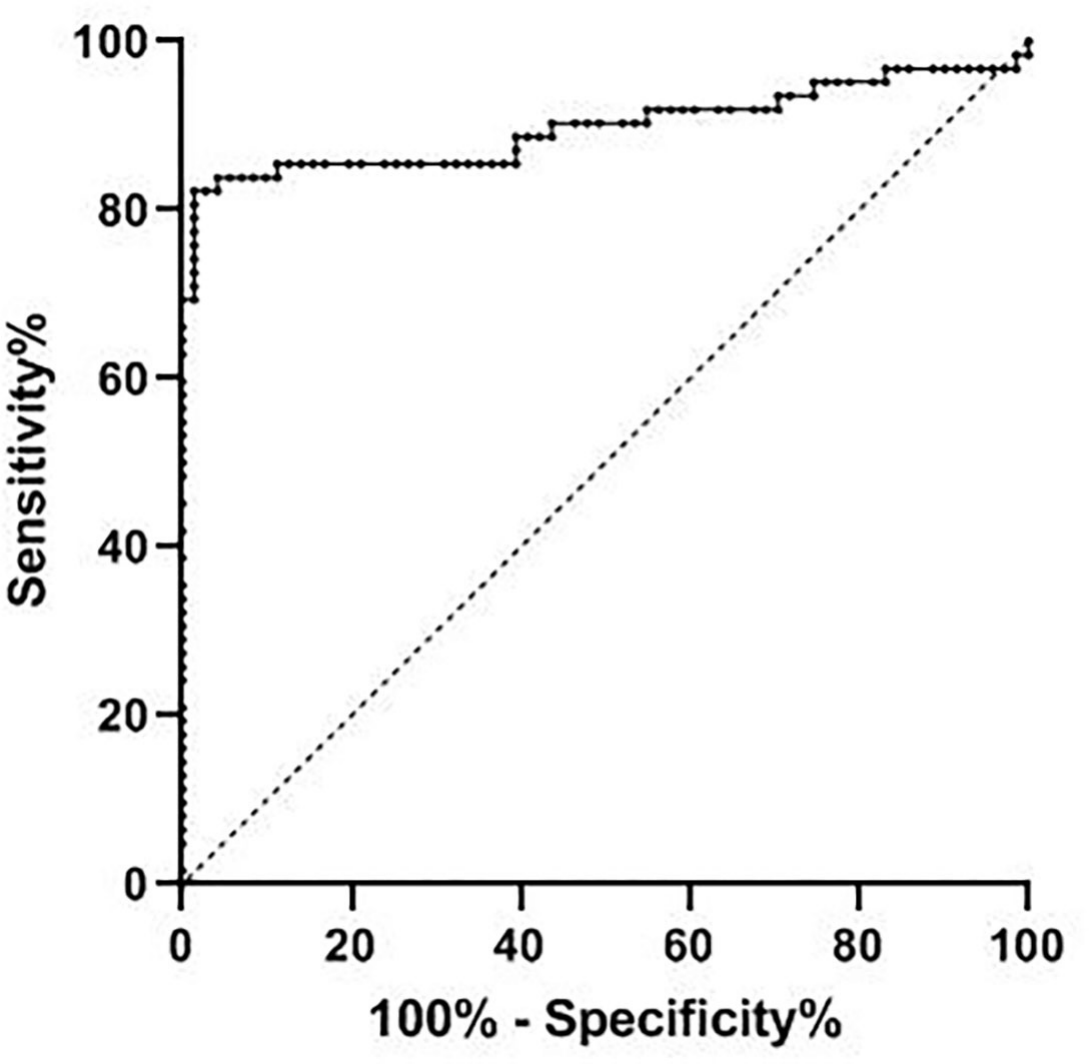
Diagnostic efficacy of CEBPA level in ovarian cancer. CEBPA, CCAAT enhancer-binding protein alpha.

The receiver operating curve analysis showed that the AUC of CEBPA level in the diagnosis of ovarian cancer was 0.898 (95% CI 0.834–0.962), the critical value was 2.36, the best cut-off value was 0.809, the sensitivity was 98.6%, and the specificity was 82.3%, as shown in Figure 3.

### Relationship Between CEBPA Level and Prognosis of Patients

**Figure 4:**
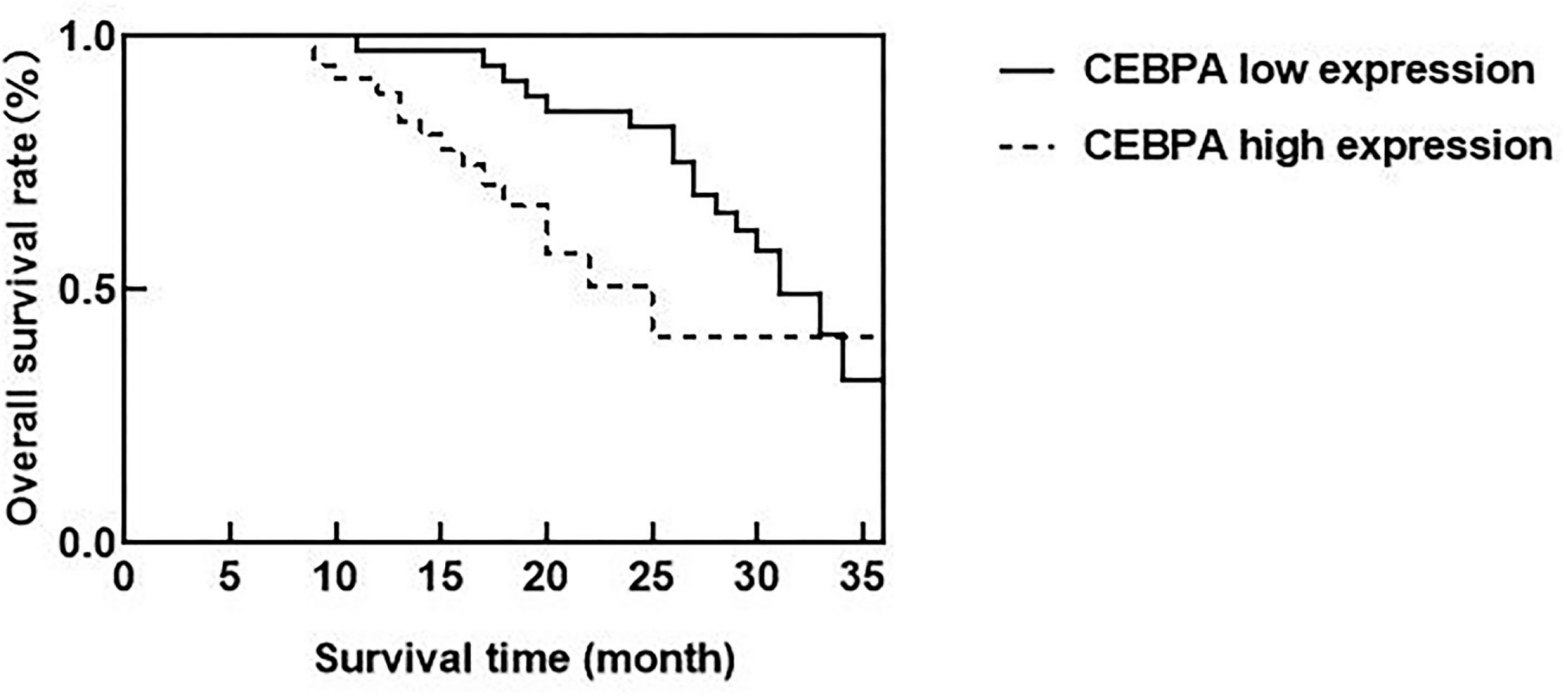
Relationship between CEBPA level and prognosis of patients. CEBPA, CCAAT enhancer-binding protein alpha.

According to the median expression of CEBPA level, ovarian cancer patients were divided into CEBPA low expression and CEBPA high expression. Ended in June 2021, all patients were followed up. Kaplan-Meier survival curve analysis showed that the median survival time of patients with CEBPA high expression was 25 months, which was lower than 31 months of patients with CEBPA low expression, and the Log-rank test was *p* = 0.034, as shown in Figure 4.

## Discussion

At present, the pathogenesis of ovarian cancer is still unknown. The pathogenesis of ovarian cancer is the result of multiple factors, which are closely related to genetic factors, hormone secretion, and the living environment (12). Due to the diversity of ovarian tissue structure and relatively complex endocrine function, the early clinical features of ovarian cancer are not obvious and lack typicality, which often leads to an extremely low detection rate of ovarian malignant tumors. Most patients are diagnosed accompanied by metastasis and spread of the abdomen and pelvic cavity, which has a negative impact on patients' life safety (13). Surgery and chemotherapy are the traditional treatments for ovarian cancer. Although they can improve patients' symptoms and prolong their life, patients are prone to drug resistance to chemotherapy, and the survival rate of patients is still not ideal and the recurrence rate is high, so the mortality rate of ovarian cancer remains high (14). Therefore, it is urgent to find reliable molecular markers.

Cytosine-cytosine-adenosine-adenosine-thymidine enhancer-binding protein alpha is the first member of the leucine zipper transcription factor family, which can affect the density and differentiation of immune cells, and is very important for inhibiting self-renewal in the hematopoietic process, cell cycle arrest, and bone marrow differentiation (15). The expression level of CEBPA is the highest in the placenta and also has a high expression level in the liver, lung, skeletal muscle, but its expression level in the brain, testis, and ovary is very low, even cannot be detected (16). CEBPA has a tissue-specific expression pattern, and its expression is upregulated during granulocyte differentiation and downregulated during monocyte transduction. CEBPA mRNA and protein have been reported in various cancer diseases (17). Chapiro et al. revealed that the upregulation of CEBPA may lead to the occurrence of precursor B-cell acute lymphoblastic leukemia, and CEBPA was activated by rearrangement with immunoglobulin gene enhancers and immunoglobulin heavy chain genes containing t(14;19)(q32;q13). CEBPA may have the characteristics of carcinogenesis and tumor inhibition in the occurrence of human leukemia (18). Lu et al. consider that CEBPA mRNA and protein levels were upregulated in some hepatocellular carcinoma (HCC) and had the activity of promoting growth in HCC cells (19). Research by Lourenço and Coffer showed that the downregulation of CEBPA expression had been confirmed in a variety of solid tumors, such as liver cancer, breast cancer, and lung cancer. CEBPA can inhibit the growth of tumors, and the inactivation of expression in these tumors was caused by promoter methylation and gene mutation (20).

Sundfeldt et al. found by immunohistochemistry that CEBPA protein was preferentially expressed in epithelial/tumor cells of ovarian cancer tissue samples, which was independent of tumor stage and grade. CEBPA was expressed in epithelial cells but not in stroma. The main expression site was the cytoplasm of cancer cells. CEBPA might be involved in the proliferation process of epithelial ovarian tumor cells *in vivo*, which could play an important role as an early molecular event (21). Yoon and Smart identified nine potential p53 binding sites in the CEBPA promoter and discovered the new role of CEBPA as a p53-regulated DNA damage-inducing gene. CEBPA was a DNA damage-inducing p53 regulatory mediator of G(1) checkpoint in keratinocytes (22). In ovarian cancer, the abnormality of p53 is common, which may affect the role of CEBPA to some extent, and then change the patient's prognosis. In addition, the development of ovarian cancer is considered to be closely related to the levels of androgen and gonadotropin, while CEBPA may control the literalization process by regulating the genes needed to maintain extensive vascular network formation of luteal cells and play an intermediary role in terminal differentiation of granulosa cells. CEBPA has involved the occurrence of ovarian cancer through gonadotropin and other related ways (23). Through real-time fluorescence quantitative PCR, WB, immunohistochemistry, and other experiments, our results showed that compared with normal ovarian and epithelial ovarian cyst patients, the expression of CEBPA mRNA and protein in patients with ovarian borderline tumor and ovarian cancer were higher, which proves that the upregulation of CEBPA may play a role in the pathogenesis of ovarian cancer. At the same time, we found that there was no correlation between the expression of CEBPA and the age, FIGO stage, histologic type, degree of histological differentiation, and lymph node metastasis of ovarian cancer patients. The occurrence of ovarian cancer may involve the changes of many proto-oncogene and tumor suppressor genes, which are related to the balance of proto-oncogene or tumor suppressor genes. The mechanism of action of CEBPA in ovarian cancer needs further study, and more large-scale studies are needed to explore the relationship between the expression level of CEBPA and the pathobiological characteristics.

The ROC curve was further constructed in this study and found that the AUC of CEBPA level in diagnosing ovarian cancer was 0.898, which indicated that CEBPA level was of high value in diagnosing ovarian cancer. The possible reason for our analysis is that CEBPA has an extensive mutation in human tumors, and the mutation rate of CEBPA in ovarian cancer may have a higher mutation rate, thus inhibiting the anti-proliferative effect of CEBPA. In addition, genome mutation or post-translational regulation/modification can reduce the effect of CEBPA. Therefore, we believe that the overexpression of CEBPA can be regarded as a diagnostic molecule in ovarian cancer patients and can be considered as a target for ovarian cancer treatment. Clinically, it is necessary to strengthen the detection of CEBPA levels in patients with ovarian lesions, so as to evaluate the disease situation of patients and formulate corresponding treatment plans according to the evaluation results, which will have a positive effect on improving the prognosis of patients. In addition, the research of Konopka et al. shows that the existence of CEBPA is positively correlated with the adverse clinical outcomes of patients, and the upregulation of CEBPA expression has a negative impact on the survival rate of patients with ovarian cancer, which can be an effective molecular marker for predicting prognosis (24). According to our results, among ovarian cancer patients, the median survival time of patients with CEBPA high expression is shorter than that of patients with CEBPA low expression, and the overexpression of CEBPA level constitutes a factor of poor prognosis of ovarian cancer patients. This may be due to the obvious carcinogenic effect of CEBPA in ovarian cancer (25). Clinicians can consider measuring the expression level of CEBPA when treating patients with ovarian cancer, which is helpful to evaluate the prognosis of patients and improve their living standards.

## Conclusion

To sum up, the expression of CEBPA mRNA and protein in patients with ovarian borderline tumor and ovarian cancer is high, and the expression of CEBPA has no obvious correlation with the pathobiological characteristics of patients with ovarian cancer, and the high expression of CEBPA has important value in the diagnosis of ovarian cancer, and it is also a poor prognostic factor of the disease. CEBPA is expected to become a new biomarker for diagnosing and evaluating the prognosis of ovarian cancer, which may play a role in the treatment choice of patients with ovarian cancer. However, the results of this study need to be verified by more patients, to further explore the specific mechanism of CEBPA expression in ovarian cancer.

## Data Availability Statement

The original contributions presented in the study are included in the article/supplementary material, further inquiries can be directed to the corresponding author.

## Ethics Statement

The studies involving human participants were reviewed and approved by the Ethics Committee of the Affiliated Hospital of Beihua University. The patients/participants provided their written informed consent to participate in this study.

## Author Contributions

LZ is responsible for the writing of the article. ML is responsible for the design of the study. ZD is responsible for the operation of the experiment. CT is responsible for the recording of the results. MF is responsible for the statistics of the data. SM is not the instructor of the whole study and is responsible for the revision of the paper. All authors contributed to the article and approved the submitted version.

## Funding

This research was supported by the 13th Five-Year Plan Science and Technology project of the Education Department of Jilin Province (JJKH20200072KJ).

## Conflict of Interest

The authors declare that the research was conducted in the absence of any commercial or financial relationships that could be construed as a potential conflict of interest.

## Publisher's Note

All claims expressed in this article are solely those of the authors and do not necessarily represent those of their affiliated organizations, or those of the publisher, the editors and the reviewers. Any product that may be evaluated in this article, or claim that may be made by its manufacturer, is not guaranteed or endorsed by the publisher.
